# Hospital Prophylaxis: The Prevention of Accidents

**Published:** 1913-12-20

**Authors:** 


### PROBLEMS OF ADMINISTRATIVE MEDICINE.

### Hospital Prophylaxis: The Prevention of Accidents.

By prophylaxis the physician understands prevention, that careful guarding against possible eventualities, which in practice is worth far more than any attempt, however successful, to cope with accidents after they have occurred. Dr. Frescolin, in a recent number of the *International Hospital Record*, has ably shown in what way such prophylactic measures, adequately carried out, can help a hospital. Accidents, we sometimes say, are bound to happen. On the contrary, where there is good management and able administration accidents ought not to happen. When they do it is a proof that there is "something rotten in the state." The avoidance of preventable accidents—and most hospital accidents are avoidable—must be the primary aim of every administrator. In order to be effective, adequate, and successful prophylaxis must be systematic and thorough.

### Poison and Lift Accidents.

Dr. Frescolin, in his article just alluded to, deals with several points on which the administrator should concentrate his attention. First, and foremost, come poisons. Now and then the risks that hospitals incur by oversight and carelessness in the matter of poisons and their administration are brought home forcibly by the publicity given to proceedings at an inquest on some patient who has by mistake taken an overdose or something not even intended for him. Indeed, only last week at the Spalding Johnson Hospital a patient died partly through the mistake of a nurse who used pure carbolic acid in mistake for a dilute solution when cleansing a girl patient's head. Poison cupboards are generally under lock and key, but in many wards strong antiseptic solutions are left unguarded, open to abuse by patients or visitors of an inquisitive mind. Is it too much to say that every poisonous solution, no matter how frequently used, shall be regarded in the same light as the solutions of the highly toxic alkaloids stored in the poison cupboard? It is all very well to say that no accidents happen. Hospital workers know that this statement is not true.

We recall the case of a patient who suddenly became acutely maniacal and was found sampling the nicely coloured carbolic solution on the dressing-table; it is an exceptional case, no doubt, but it points a useful moral. The same patient nearly succeeded in throwing himself out of a window, which raises the question of the prophylaxis of accidents to patients in floors that have unguarded windows and stairways. In children's wards especially all windows should be guarded if they look flush into the street below, just as all staircases are guarded by railing or netting. The latter point is always attended to; the window-guards are almost invariably forgotten. Lifts are another source of accidents. The automatic elevator is practically fool and patient proof, but elder institutions which do not possess it run grave risks in allowing lift doors to be unguarded by nets. Lifts and other openings which may cause falls should invariably be under close supervision, and it is probable that the extra expense entailed in having special lift attendants amply repays itself in the long run with regard to equipment. To put unguarded china utensils in a patient's bed is a mistake which has led to disaster before now. Unguarded hot-water bottles have been the means of landing at least one institution in the law courts. China bed-pans are now rarely used; the enamel-ware pan serves better, is cheaper, lighter, and unbreakable. But some institutions still use the dangerous old-fashioned china pan without even taking necessary precautions. It is needless to say that precautions must be taken whenever a breakable instrument is used on a patient. Nurses are supposed to be specially trained in the use of thermometers, catheters, and enemata tubes, but even with such training accidents sometimes happen. Only by scrupulous carefulness and gentleness can they be prevented, and the necessity for such care should be impressed upon every hospital worker.

### Anæsthetic Fatalities.

Indeed prophylaxis, in the sense we are using the term, is as necessary for the highly trained staff as for the attendant administrative staff. Take, for example, the administration of anæsthetics. It is held by many competent anæsthetists that fatalities under an anæsthetic are preventable in the majority of cases. It is a professional fashion of the day to ascribe many of them to "lymphatism," but both this rather vague clinical entity and "delayed chloroform poisoning"—which to an extent is certainly preventable—are bogeys which at present bear the sins, both of commission and omission, of unskilful administration by careless anæsthetists. We do not think we put the case harshly and without sympathy for the administrator who, once in a while, finds himself up against a really bad case in which no human skill can avert disaster. But we do say that a large number of fatalities under anæsthetics could have been, and ought to have been, avoided had the administrator taken proper precautions, meticulous or not, to safeguard his patient. Post-anæsthetic pneumonia, vomiting, and spasm are complications which may occur even with all due precautions to avoid them. But they occur so frequently that the inference is that these precautions have not been adequate, since it has been fully shown that with due care they may, in the vast majority of cases, be avoided. This chapter of the question is one which every member of the staff, both in his own and in institutional interests, ought to study most carefully. Few better introductions to the study can be found for medical men than the thoughtful articles—classics of surgical literature as they are—"Fatalities in Surgery," by Sir James Paget, and "Surgical Luck and Disaster," by Pirogoff.

An interesting point is raised by Dr. Frescolin in regard to the prevention of disease among the staff. All hospital workers are exposed to serious dangers of infection, which are doubled through fatigue entailed by overwork. The toll exacted annually from students and demonstrators is larger than the public realises; it is equally heavy among the nurses. Careful supervision of the work, modern up-to-date labour-saving devices, proper surroundings, and, above all, adequate provision for sanitation will do much to decrease that toll, but are they enough? Dr. Frescolin thinks not. His suggestion is that more active prophylactic measures should be employed.

### Mistaken Heroics.

Thus he suggests that preventive inoculation should be more extensively employed than it is now, that no one ought to be allowed to work in a modern hospital unless he, or she, has been "immunised" against smallpox and typhoid. This is a counsel of perfection, and there are many practical difficulties in the way of carrying it out. But we may at least go so far as to say that no one should be given hospital work who is liable, through inherent constitutional defects, to suffer through that work or to be a possible cause of mischief, unconsciously, to others. These provisions are sometimes overlooked. We have known surgeons suffering from pulmonary phthisis continue their ward work, to the admiration of their colleagues, who think that such self-sacrifice is heroic. It is nothing of the kind. A school-teacher in such a condition would be urged to give up the work, not for his own sake alone, but for the sake of his pupils. Is it wise, or fair, that the surgeon should continue to work when there is a possibility, however remote, of infecting his patients? The question presents undoubted difficulties, which hospital committees are chary of tackling for various reasons, but it is a question that ought to be faced if hospital prophylaxis is meant to be a real safeguard to all concerned.

